# A technique to retrieve an internalised embedded central venous catheter

**DOI:** 10.1186/s42155-024-00436-8

**Published:** 2024-02-26

**Authors:** John Finnegan, Pradeep Govender

**Affiliations:** Department of Radiology, Tallaght University Hospital, Dublin, Ireland

**Keywords:** Venous intervention, Dialysis access, Central venous access

## Abstract

**Background:**

Central venous catheters may become embedded due to the formation of adhesions between the indwelling catheter and the vein wall.

**Case presentation:**

A 49-year-old patient with bacteraemia was referred for retrieval of an embedded internalised central venous dialysis catheter. Recently the catheter had been surgically ligated at the venotomy site internalising the intravascular catheter component, which precluded antegrade ballooning through the catheter hub.

Seldinger technique was used to access the catheter lumen within the left internal jugular vein and through and through access was established across the catheter. Retrograde endoluminal balloon dilation was performed to disrupt adhesions and free the catheter. The catheter was snared over the wire and removed from the right femoral vein.

**Conclusion:**

This case report outlines an effective, minimally invasive retrieval method in a rare case of an embedded internalised central venous catheter.

**Graphical Abstract:**

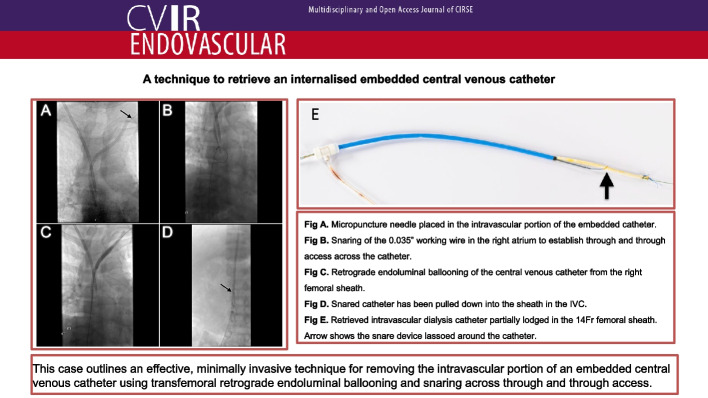

**Supplementary Information:**

The online version contains supplementary material available at 10.1186/s42155-024-00436-8.

## Background

Tunnelled central venous catheters (CVC) are usually easily removed with gentle traction after blunt dissection and release of the subcutaneous fixation cuff. A stuck or embedded tunnelled catheter is a complication that can occur due to the formation of a fibrin sheath or adhesions between the vein and the indwelling catheter. Antegrade endoluminal balloon dilation is an important minimally invasive endovascular technique for removing embedded catheters.

## Case presentation

A 49-year-old patient with end stage renal disease and a left internal jugular tunnelled dialysis catheter presented with bacteraemia. The dialysis catheter which had been inserted 7 years ago remained firmly embedded despite traction. Initially the patient was referred to the surgical team who attempted to remove the catheter in the operating theatre. In an attempt to apply direct traction to the catheter an incision was made at the base of the neck. Here the catheter was exposed but despite direct traction the catheter remained firmly stuck. The catheter was ligated at the venotomy site, internalising the intravascular portion of the catheter while the tunnelled and external portions of the catheter were removed. A temporary non tunnelled right internal jugular venous catheter was placed, the patient was transferred to our institution and referred to IR for retrieval.

**Fig. 1:**
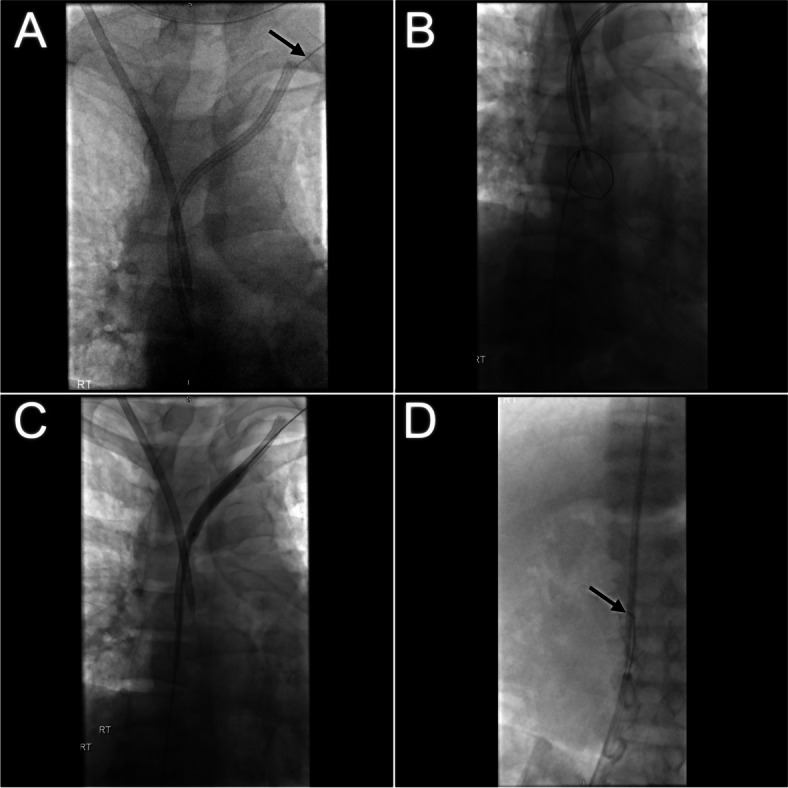
**a** Micropuncture needle positioned in the intravascular portion of the embedded catheter in the proximal left brachiocephalic vein (arrow). Temporary right internal jugular central venous catheter in situ. **b** Snaring of the 0.035″ working wire in the right atrium. **c** Retrograde endoluminal ballooning of the central venous catheter from the right femoral sheath. **d** The freed catheter has been snared and drawn into the long femoral sheath in the IVC (arrow)

Informed consent was obtained. Under light sedation, using ultrasound and fluoroscopic guidance a S-Mak micro puncture kit (Merit Medical, Utah) was used to access the lumen of the stuck dialysis catheter at the confluence of the distal left internal jugular vein and the brachiocephalic vein (Fig. 1a). The 5Fr micro sheath was placed over the microwire into the catheter. A 250 cm 0.035″ Advantage wire (Terumo, New Jersey) was advanced through the lumen of the catheter into the right atrium. Next, a 30 cm 14Fr Performer sheath (Cook Medical, Indiana) was placed in the right femoral vein. The wire was snared in the right atrium using an Amplatz Goose neck snare (eV3 Inc, Minnesota) (Fig. 1b) to establish through and through access across the catheter between the left neck and right groin.

**Fig. 2:**
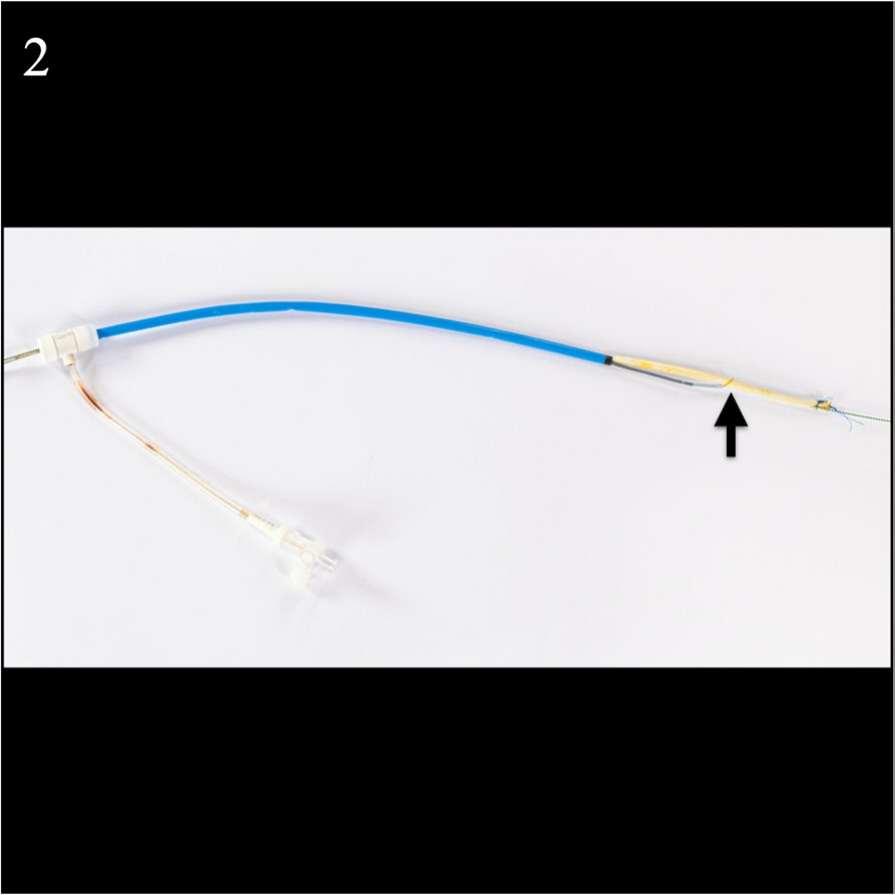
Retrieved intravascular dialysis catheter partially lodged in the 14Fr femoral sheath. The snare device is securely lassoed around the mid portion of the catheter (arrow)

From the right femoral sheath a 6 mm x 40 mm and then a 8 mm x 40 mm balloon Powerflex Pro PTA balloon (Cordis, Florida) was advanced over the wire into the catheter and retrograde endoluminal dilation was performed along the length of the catheter (Fig. 1c). This effectively disrupted the fibrin sheath and adhesions to free the catheter. From the right femoral sheath the catheter was snared over the wire. Under gentle traction the catheter was easily drawn down into the IVC and partially into the sheath (Fig. 1d). The catheter and sheath were removed in tandem from the right femoral vein over the wire (Fig. 2).

**Fig. 3:**
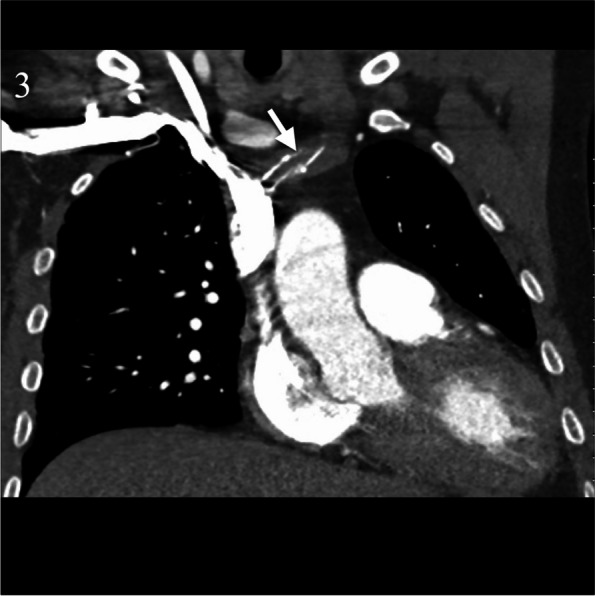
CTPA 6 months later incidentally demonstrates a heavily calcified fibrin sheath (arrow) traversing the left brachiocephalic vein in which the catheter had been embedded

A CTPA performed 8 months later showed the heavily calcified fibrin sheath traversing the brachiocephalic vein in which catheter had been embedded (Fig. 3).

## Discussion

This case outlines an effective, minimally invasive technique for removing the intravascular portion of an embedded CVC using transfemoral retrograde endoluminal ballooning and snaring across through and through access.

In a large retrospective study of 2048 haemodialysis catheters inserted only 19 (0.92%) required endovascular or surgical removal [1]. Factors such as a long catheter dwell time (defined as > 2 years) and the presence of central vein stenosis correlate with an increased risk of catheters becoming embedded [2].

Several IR techniques exist for removing embedded catheters. Endoluminal balloon dilation first described by Hong in 2011 is accepted as an effective technique for removing stuck catheters. Hong described making an incision in the neck at the venous entry point and exposing the catheter to allow antegrade endoluminal balloon dilation [3]. Endoluminal balloon dilation works by stretching the fibrin sheath and adhesions between the catheter and the vein allowing the catheter to be removed under traction. A modification of this technique uses low profile balloons inserted directly through the external catheter hub which averts the need for a soft tissue dissection [4]. Devices such as peel away sheaths, laser sheaths and Vollmar rings have also been successfully used to directly strip the catheter from the vein wall [5–7]. These methods are more invasive and although data regarding safety data is limited to case series there is conceivably a higher risk of venous injury. Abandoning an embedded catheter is a last resort. Abandoned catheters can pose a risk of future bacteraemia or cause central venous obstruction which is a significant problem for patients with end stage renal disease, many of whom are reliant on patent central veins for haemodialysis.

## Conclusion

This case report outlines an effective, minimally invasive technique to retrieve the intravascular portion of an embedded central venous catheter. The retrograde endovascular retrieval approach described in this article was necessary as the external and tunneled portions of the catheter had been surgically removed, which precluded antegrade endoluminal ballooning through the external catheter hub. Through and through access facilitated easy retrograde ballooning and snaring of the catheter, while ensuring stability of the freed catheter and maintaining access across the thoracic central veins in case of venous injury.

### Supplementary Information

**Supplementary Material 1.**

## Data Availability

Non applicable.

## Abbreviations

CVC: Central venous catheter
ESRD: End stage renal disease
